# Draft Genome Sequence of the Griseofulvin-Producing Fungus Xylaria flabelliformis Strain G536

**DOI:** 10.1128/MRA.00890-19

**Published:** 2019-09-19

**Authors:** Matthew E. Mead, Huzefa A. Raja, Jacob L. Steenwyk, Sonja L. Knowles, Nicholas H. Oberlies, Antonis Rokas

**Affiliations:** a Department of Biological Sciences, Vanderbilt University, Nashville, Tennessee, USA; b Department of Chemistry and Biochemistry, University of North Carolina at Greensboro, Greensboro, North Carolina, USA; University of California, Riverside

## Abstract

The draft genome of the ascomycete fungus Xylaria flabelliformis (previously known as Xylaria cubensis) was sequenced using Illumina paired-end technology. The assembled genome is 41.2 Mb long and contains 11,404 genes. This genome will contribute to our understanding of *X. flabelliformis* secondary metabolism and the organism’s ability to live as a decomposer as well as an endosymbiont.

## ANNOUNCEMENT

The genomes of fungi in the order Xylariales (Sordariomycetes, Ascomycota) contain some of the highest numbers of genes involved in secondary metabolism among fungi (1). Xylaria flabelliformis (previously known as Xylaria cubensis) (2, 3) is a filamentous fungus in the order Xylariales that lives both as a decomposer of organic matter (4) and as an endosymbiont of plants and lichens (5). *Xylaria flabelliformis* is known to produce the fungistatic compound griseofulvin, an FDA-approved drug that is also considered an “essential medicine” by the World Health Organization (6, 7). To better understand the secondary metabolism and evolution of *X. flabelliformis*, we sequenced the genome of a representative strain.

*Xylaria flabelliformis* strain G536 (8) was grown on liquid yeast extract soy peptone dextrose (YESD) medium. After 7 days, the mycelium was filtered through a sterile filter, retrieving the mycelial mass, which was then ground to a fine powder with a sterile mortar and pestle by using liquid nitrogen. The fine powder was then transferred to a bashing bead tube with DNA lysis buffer from the Zymo Quick-DNA fungal/bacterial miniprep kit (catalog number D6005). The powder in the bashing bead tube was further disrupted and homogenized in a Qiagen TissueLyser LT bead mill for 5 min. Genomic DNA was extracted using procedures outlined in the Zymo Quick-DNA fungal/bacterial miniprep kit and sonicated to a size of ∼550 bp. A sequencing library was constructed using the Illumina TruSeq library preparation method. Paired-end sequencing (300 bp from each end) was performed on an Illumina MiSeq version 3 instrument run at HudsonAlpha Discovery (Huntsville, AL), producing a total of 23,234,771 paired-end reads.

The raw reads were trimmed of adapter and low-quality sequences with Trimmomatic version 0.36 (9) by using a custom list of adapter sequences (see the Figshare document at https://www.doi.org/10.6084/m9.figshare.8986505) and the parameters “ILLUMINACLIP:2:30:10 LEADING:3 TRAILING:10 SLIDINGWINDOW:4:15 MINLEN:50.” The reads were then *de novo* assembled with SPAdes version 3.13.1 (10) using the “–careful” and “–cov-cutoff auto” options. The final genome assembly consisted of 41,150,291 bp spread over 155 scaffolds (164 contigs), with an *N*_50_ value of 488,275 bp and a GC content of 47.44%. Gene prediction was performed using AUGUSTUS version 3.3.2 (11), with Histoplasma capsulatum as the training species and the settings “–minexonintronprob=0.1,” “–minmeanexonintronprob=0.4,” and “–noInFrameStop=True.” The genome contained 11,404 predicted protein-coding genes. Analyses of the predicted protein product sequences with BUSCO version 3.1.0 (12) and the sodariomycete_odb9 database showed that the proteome contained 93.6% of the BUSCOs as complete proteins. The annotation was converted to an SQN file by using the NCBI-provided script tbl2asn and submitted to GenBank.

To gain insights into *X. flabelliformis* secondary metabolism, we predicted biosynthetic gene clusters with antiSMASH version 4.1.0 (13) using the “–taxon fungi” option. A total of 86 putative biosynthetic gene clusters were predicted, including clusters likely to produce both griseofulvin and cytochalasin, two metabolites known to be produced by *X. flabelliformis* G536 (8, 14). Tables summarizing the antiSMASH results can be found in the Figshare document at https://www.doi.org/10.6084/m9.figshare.8986505. Our work represents a major step toward understanding the evolution and biochemical output of this fungus.

### Data availability.

This whole-genome shotgun project has been deposited at DDBJ/ENA/GenBank under the accession number VFLP00000000. The version described in this paper is VFLP01000000. The Illumina raw reads have been deposited at the Sequence Read Archive under accession number SRX5939388.
